# Examining decisional needs and contextual factors influencing fertility status assessment among young female survivors of childhood cancer: A sequential mixed methods study protocol

**DOI:** 10.1371/journal.pone.0286511

**Published:** 2023-06-14

**Authors:** Brooke Cherven, Nataliya V. Ivankova, Jessica B. Spencer, Anne M. Fitzpatrick, Karen C. Burns, Jenna Demedis, Holly R. Hoefgen, Ann C. Mertens, James L. Klosky

**Affiliations:** 1 Aflac Cancer and Blood Disorders Center at Children’s Healthcare of Atlanta, Atlanta, GA, United States of America; 2 Department of Pediatrics, Emory University School of Medicine, Atlanta, GA, United States of America; 3 Department of Health Services Administration, University of Alabama at Birmingham, Birmingham, AL, United States of America; 4 Department of Gynecology and Obstetrics, Emory University School of Medicine, Atlanta, GA, United States of America; 5 University of Cincinnati College of Medicine, Cincinnati, OH, United States of America; 6 Cincinnati Children’s Hospital Medical Center, Cincinnati, OH, United States of America; 7 Center for Cancer and Blood Disorders at Children’s Hospital Colorado, Aurora, CO, United States of America; 8 Department of Pediatrics, University of Colorado School of Medicine, Aurora, CO, United States of America; 9 Washington University School of Medicine, St. Louis, MO, United States of America; PLoS ONE, UNITED STATES

## Abstract

**Introduction:**

Female cancer survivors who received gonadotoxic cancer treatment are at risk for profound diminished ovarian reserve and/or primary ovarian insufficiency with resulting infertility, which can be associated with distress and decreased quality of life.. Despite prioritizing future parenthood, many survivors are unsure of the impact of their treatment on their future fertility, and little is known about the perceived reproductive health needs and factors associated with receipt of a fertility status assessment (FSA). There is a lack of developmentally appropriate reproductive health decisional support interventions available for emerging adult cancer survivors. This study will explore the perceived reproductive health needs of emerging adult female survivors of childhood cancer and to identify decisional and contextual factors that influence pursuit of FSA using an explanatory sequential quantitative to qualitative mixed methods design.

**Methods and analysis:**

This study will enroll 325 female survivors (aged 18 to 29 years and >1-year post treatment; diagnosed with cancer < age 21 years) from four cancer centers in the United States. Sociodemographic and developmental factors, reproductive knowledge and values, decisional needs, and receipt of an FSA will be assessed through a web-based survey. Informed by survey findings, a subset of participants will be recruited for qualitative interviews to explore decisional factors associated with uptake of an FSA. Clinical data will be abstracted from the medical records. Multivariable logistic regression models will be developed to identify factors associated with FSA and qualitative descriptive analysis will be used to develop themes from the interviews. Quantitative and qualitative findings will be merged using a joint display to develop integrated study conclusions and direct future interventional research.

## Introduction

Cancer treatment has improved such that survival rates overall for childhood and adolescent cancer now surpass 80% [1]. There are nearly 400,000 survivors of childhood/adolescent cancers in the United States, most of whom are now young adults and in their reproductive years [2]. Survivors who received gonadotoxic therapies are at risk for cancer treatment-related infertility [3, 4]. Infertility rates range from 11–26% among young adult female cancer survivors [5] and when compared with healthy siblings, female survivors are more likely to experience infertility [6] and less likely to report a pregnancy [7]. A large proportion of female survivors of childhood cancer receive gonadotoxic treatment, representing a population who may be at risk for a shortened reproductive window and potentially interested in fertility preservation post cancer treatment [8, 9].

The ability to conceive and have children is a priority among patients and families from the time of cancer diagnosis [10] into long-term survivorship [11–13]. Across multiple studies of female survivors of childhood cancer, >75% report a desire for children in the future [12, 14, 15]. Potential infertility is a substantial source of distress for young adult cancer survivors and has been associated with depression, anxiety, stress, and trauma [16–18]. Uncertainty about their own gonadal function and risk for infertility is reported by 48–77% of survivors of childhood cancer, despite their interest in biological children, underscoring the need for interventions to increase uptake of gonadal assessments among interested survivors [19–21]. In an earlier study, over half of 179 female cancer survivors (mean age 29 years) reported unmet information needs regarding their options to assess and preserve fertility during survivorship. Unmet needs were associated with greater decisional conflict (i.e., feeling conflicted about a course of action) regarding fertility preservation post cancer treatment, while the receipt of a fertility evaluation was related to lower decisional conflict [22].

There are no definitive tests that predict fertility among cancer survivors, as fertility can be affected by various factors which are unrelated to previous gonadotoxic treatments including the partner’s health, sperm quality/quantity, health problems that impact fecundity, lifestyle/behavioral factors, and others. Assessing the risk for infertility among cancer survivors includes consideration of prior gonadotoxic exposures and other medical history, gonadal assessment, and counseling regarding family building options, which can broadly be described as a fertility status assessment (FSA). An FSA for females typically includes a medical history, assessment of menstrual-cycle specific laboratory hormones and a pelvic ultrasound with antral follicle count. This testing should be followed by discussion of ovarian reserve and the risk of infertility and/or premature ovarian insufficiency, and available treatment options. These consults are typically completed by reproductive endocrinologists [23]. Among survivors who received gonadotoxic cancer treatments, a consultation and FSA is recommended in accordance with national guidelines among interested patients [24]. Prior studies demonstrate that young female cancer survivors are interested in an FSA and receiving further reproductive health counseling [25, 26]. Females who are at risk for premature ovarian insufficiency may have a shortened window for fertility preservation, therefore timely assessment provides an opportunity for discussing options for fertility preservation, and potentially intervening in a timely fashion in order to improve preservation outcomes. However, little is known regarding factors that influence uptake of an FSA in female survivors of childhood cancer.

Reproductive health and consideration of FSA is particularly relevant to the emerging adult population. This is because emerging adulthood (18–25 years of age) represents a developmental period of transition from adolescence to adulthood [27]. Emerging adults are diverse in their level of independence and achievement of milestones, including educational attainment, employment, and relationship status, which may impact a survivor’s pursuit of an FSA [27, 28]. Psychologically, emerging adulthood is a time of experiencing possibilities, exploration, and feeling ‘in between’ childhood and adulthood [27]. During emerging adulthood, cancer survivors may be newly accessing reproductive health services, forming committed romantic relationships, and navigating the expectations of friends and family members regarding family-building. In this developmental context, emerging adulthood may be a time when survivors are anxious to explore their options for biological parenthood through an FSA.

### Study purpose

While potential infertility is a top concern among young cancer survivors, little is known about the perceived reproductive health needs and factors associated with uptake of a FSA among female cancer survivors. The purpose of this two-phase sequential quantitative to qualitative mixed methods study is to explore the perceived reproductive health needs of female cancer survivors and to identify decisional and contextual factors that influence receipt of an FSA. The goal of the first, quantitative study phase is to establish a knowledge base of the reproductive health needs of this population and identify sociodemographic, developmental, and reproductive-health factors that are related to receipt of an FSA. This will be achieved by surveying 325 survivors recruited from four cancer centers across the United States. The goal of the second, qualitative phase, is to build on the quantitative results and elucidate patient experiences with FSA and preferences for a decisional support intervention by interviewing 32 survey participants purposefully selected based on their reported receipt/interest in an FSA. The rationale for integrating quantitative and qualitative methods in this study is to expand our understanding of the factors that influence survivors’ receipt of reproductive health services and to inform the development of a decisional support intervention for survivors interested in an FSA. Quantitative, qualitative, and mixed methods research questions to guide the study are:

**Quantitative:** What are the reproductive health values, behaviors, and knowledge gaps of young adult female survivors of childhood cancer? Which sociodemographic, developmental, and reproductive health factors are related to receipt of an FSA?

**Qualitative:** How do contextual factors influence female cancer survivors’ decision to pursue or not pursue an FSA? What are barriers or facilitators to accessing an FSA and/or reproductive health services?

**Mixed Methods:** What components, grounded by the quantitative and qualitative results, should be incorporated into reproductive health and decisional support interventions to address barriers and increase decisional satisfaction for young adult females considering an FSA?

## Methods and analysis

### Study design

While there have been independent quantitative and qualitative investigations in this area, a mixed methods approach is necessary to fully understand survivor perspectives regarding reproductive health and FSA, with a focus toward future intervention development and testing. Mixed methods research involves the collection of both quantitative and qualitative data, with a key feature of integrating findings to determine meta-inferences or integrated study conclusions [29]. A two-phase mixed methods study design will enhance assessment of the developmental factors, psychological and cultural considerations, and provide insight into participants’ experiences in accessing an FSA. The depth of the qualitative data will complement the quantitative data, minimizing the weaknesses in the use of each approach in the study [30]. Using pragmatic philosophical lens, this study will combine and mix data to represent multiple perspectives which will contribute to both a broader and deeper understanding [31, 32]. Results will guide the development of a decisional support intervention and identify additional areas of focus for reproductive health research.

**Fig 1:**
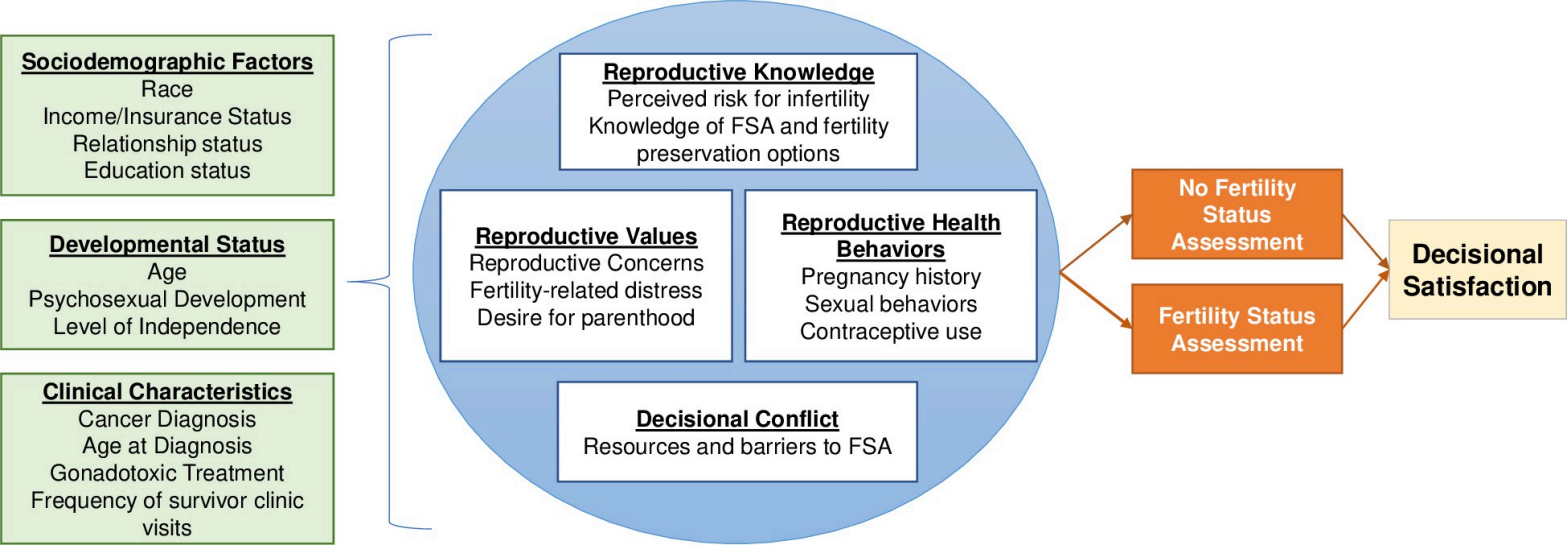
Study conceptual model.

**Fig 2:**
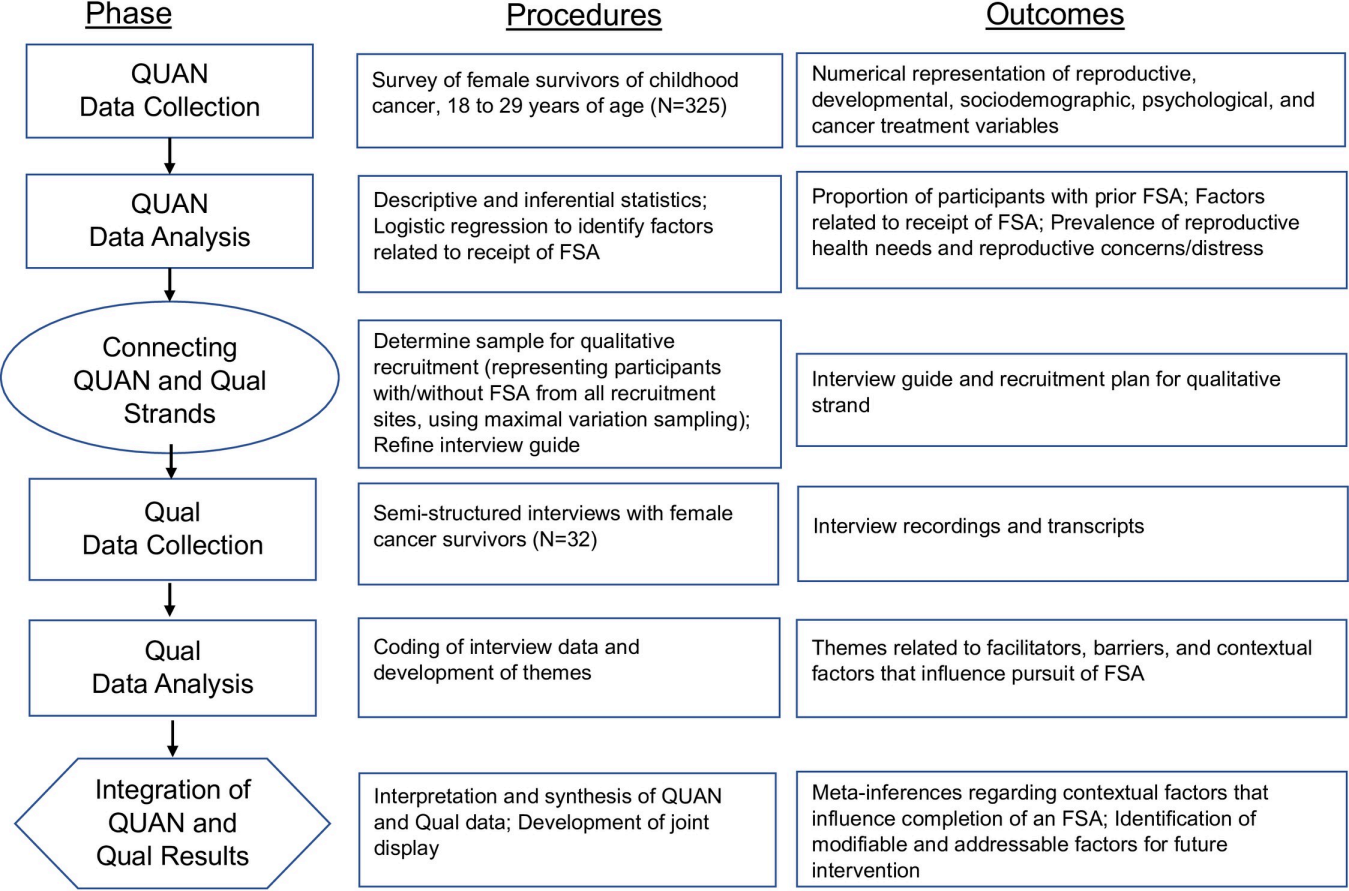
Sequential mixed methods procedural diagram. Sequential QUAN→ Qual mixed methods procedural diagram (QUAN = quantitative; Qual = Qualitative; FSA = fertility status assessment; figure adapted from Ivankova, Creswell, & Stick, 2006 [30]).

This study is informed by the Ottawa Decision Support Framework that purports an individual’s decisional needs will affect decision quality, behavior, and emotions regarding the decision [33]. The conceptual model, including concepts of interest, is presented in Fig 1. The timing of data collection is sequential, with quantitative data collection and analysis conducted first to inform the sample and data collection for the subsequent qualitative phase. Quantitative data is weighted more heavily to align with the primary aim of identifying reproductive health needs and decisional factors amongst a large, representative sample. The quantitative phase includes strategies to enhance generalizability of the findings, described later under Quality Assurance, and utilizes prior research in the field to inform quantitative measurement. Additionally, the study is centered around receipt of FSA, which is an outcome that will be measured through the quantitative phase. Findings from the qualitative phase will expand on survivors’ experiences with reproductive health and FSA to inform intervention development. The procedures and points of integration (connecting phases and integrated analysis) are included in Fig 2.

### Study setting

The population of interest for this study is female survivors of childhood cancer, 18 through 29 years of age. A sample of 325 survivors will be identified and recruited from four cancer centers throughout the United States. The participating sites have established fertility clinical services, are active members of the Children’s Oncology Group, and have a history of collaboration through the Pediatric Initiative Network of the Oncofertility Consortium [34]. These sites were selected to represent a geographically diverse population of cancer survivors and have established fertility programs where they refer cancer survivors for FSA (outcome of interest). Fertility programs are not uniformly present across cancer centers [35], therefore it was important to choose sites where participants would have the opportunity to pursue an FSA. All sites completed a site selection survey, reporting their estimated number of eligible patients and establishing the process for which they would identify eligible participants (using site specific registries/databases etc.).

Participants are eligible for this study if they are (1) 18.00 to 29.99 years of age, (2) diagnosis of cancer < 21.00 years of age, (3) female sex, and (4) received gonadotoxic cancer treatment (e.g., alkylating chemotherapy agents, heavy metal chemotherapy, radiation including the gonads, cranial radiation ≥ 30 Gy, or hematopoietic cell transplant) [4]. Participants are excluded if they (1) have cognitive dysfunction such that they would be unable to complete the survey, or (2) bilateral oophorectomy. Participants who speak English or Spanish are eligible to complete the quantitative survey. Recruitment is stratified across age groups, with emphasis on the early years of emerging adulthood to ensure a developmentally-diverse sample; with a goal of recruiting two-thirds of the sample from participants 18.00–24.99 years of age (n = 215) and one-third from participants 25.00–29.99 years of age (n = 110).

The primary endpoint for this study is receipt of an FSA (measured as yes vs. no/I don’t know). Based on insights from the literature, and our own clinical experiences, we anticipate 20% (n = 65) of the sample will have received an FSA and 260 respondents will have not. This sample size, obtained by sampling 4 study sites, achieves 80% statistical power to detect an odds ratio of 2.4 for discrete exposures. This odds ratio corresponds to 70.4% of respondents in the FSA group having the exposure, versus 50% of respondents in the non-FSA group having the exposure (i.e., a percent difference of 20.4%). Power was calculated in PASS v.14.0.8 (Kaysville, UT), with a two-sided un-pooled Z-test, an intra-cluster correlation (ICC) of 0.01, and a statistical significance level of 0.05.

### Sampling

#### Quantitative phase

This study will use a probability approach to sampling for the quantitative phase to maximize the representativeness of the study sample. The participating study teams will identify eligible patients using site-specific databases, cancer registry data, or similar methods. Each site then randomizes their list of eligible patients and consecutively recruits through email/phone call until recruitment goals are reached for their site; survivors may also be approached during in-person clinical visits. Prospective participants then receive a link to complete the web-based REDCap survey; consent for participation is obtained at the beginning of the survey. Participants receive a $10 gift card for completing the survey.

#### Qualitative phase

Participants for the qualitative interviews will be recruited purposively from those who indicated on the survey that they (1) would be willing to take part in a qualitative interview and (2) responded to the survey item assessing receipt of an FSA. The interviewers for this study are English-speaking, therefore only participants who speak English will be invited to take part in the qualitative interview. We will use a qualitative descriptive approach, which is low-inference and seeks to describe the experiences of participants through their words and experiences [36]. In line with this approach, we will use maximum variation sampling, which is a preferred method for descriptive qualitative inquiry [37, 38]. The participants will be varied across each of the four participating sites and by receipt of FSA. Interviews will be conducted until data saturation is reached; we estimate the sample size to be 32 participants. Participants will receive a $30 e-gift card for completing an interview.

### Data collection

#### Quantitative phase

Data for the quantitative phase will be collected through a web-based survey assessing sociodemographic and developmental factors, reproductive factors, psychological and decisional factors, and history of FSA. Sociodemographic factors include gender identity, race/ethnicity, religion, education level, household income, insurance and relationship status, and items are consistent with national surveys of young adults [39]. Developmental factors will be assessed through the lens of emerging adulthood using The Markers of Adulthood (MoA) [28, 40] and the IDEA-8 [41] which have been used in large multi-site studies with emerging adults across diverse populations [42]. We will assess reproductive knowledge regarding cancer and fertility preservation [43], fertility after cancer (e.g., impact of cancer treatment on fertility, knowledge of infertility testing and treatment) [44, 45], and survivors’ perceived risk for infertility compared with peers who have not had cancer [21]. Reproductive values will be assessed using the modified Reproductive Concerns Scale (mRCS), which has been validated among adolescent and young adult survivors of childhood cancer [46, 47], the Fertility Problem Inventory Short Form [48, 49], and the Couple’s Relationship Concern subscale from the Reproductive Concerns After Cancer scale [50, 51].

Reproductive health behaviors include pregnancy and birth history, contraceptive history and current use, and sexual behaviors [39, 52]. Reproductive health needs will be assessed through items identifying unmet educational needs, contraceptive use and patterns, and interest in FSA. Decisional conflict will be measured using the Decisional Conflict Scale, and decisional satisfaction among participants who have received an FSA will be measured using the Effective Decision Subscale [53]. Participants who respond ‘Yes’ to the Fertility Status Assessment item will also be asked details about this assessment (e.g., testing, results, discussion of infertility treatment options). Participants who respond ‘No’ or ‘I don’t know’ will be asked their level of interest for completing an FSA and perceived barriers to not receiving an FSA (e.g., lack of awareness, cost) [54].

Clinical characteristics, including cancer diagnosis, date of diagnosis, number of cancer survivor clinical encounters, gonadotoxic therapuetic exposures [alkylating and heavy metal chemotherapy, gonadoxic radiation], surgeries, history of hematopoietic cell transplant, and date of cancer treatment completion will be abstracted from participant’s medical records using an adapted version of the Children’s Oncology Group Summary of Cancer Treatment Template [24].

#### Qualitative phase

Data for the qualitative phase will be collected through semi-structured interviews. Participants will be contacted and invited to complete a 45-minute semi-structured interview over the phone or using Zoom web conferencing (HIPAA compliant) with a trained qualitative interviewer. Consistent with a sequential design, the interview guide will be developed and informed by descriptive results from the quantitative phase [30]. We anticipate that the interviews will include a discussion of factors that influenced participants’ awareness of FSA options, decision to pursue or not pursue FSA, satisfaction with that decision, barriers encountered when accessing FSA, and resources needed to support survivors who are interested in FSA.

### Study timeline

Study recruitment and quantitative data collection began April 4^th^, 2022 at the coordinating center, with all participating sites actively recruiting by January 11, 2023. Qualitative data collection is planned to begin in summer 2023.

### Data analysis

#### Quantitative phase

Descriptive statistics will be examined using means and standard deviations, medians and interquartile ranges, or percentages and frequencies, as appropriate. For the primary outcome variable (i.e., “yes” for an FSA), multivariable logistic regression models will be developed to identify factors related to receipt of an FSA. Final multivariable results will be guided by the bivariable associations and determined using backward selection procedures, as well as any clinically relevant variables.

#### Qualitative phase

Interviews will be audio-recorded and professionally transcribed verbatim and checked for accuracy [55]. Inductive thematic analysis will be used, which involves searching for patterns and meanings in the data by systematically organizing the data into categories and themes from specific to general. Node reports (e.g., text associated with a specific code) will be generated by the research team members with expertise in qualitative research, using NVivo (v12) to facilitate identification of sub-themes and similarities and differences by group (i.e., site and receipt of FSA, as well as sociodemographic factors) [56]. Final themes and sub-themes will be presented and agreed upon within the research team.

### Quality assurance

To assure quality in this mixed methods study, we will use strategies associated with the quantitative phase, qualitative phase, and strategies that support mixed methods meta-inferences [57, 58]. Strategies to enhance quality in the quantitative phase include using a random sampling approach to increase generalizability and external validity of the findings to the larger population of interest. We determined our sample size after conducting a power analysis to ensure we would be able to detect clinical meaningful differences between survivors who had and had not received an FSA. We have also chosen measures for the survey that are validated in the participant age range to increase internal validity. Finally, all study staff have undergone training to ensure fidelity to the protocol across sites [59]. Data will be managed using REDCap [60] and a data management plan will be used throughout the study to track data collection and ensure adherence to the study protocol. Upon study completion, data will be available with request.

Strategies to enhance credibility and trustworthiness for the qualitative phase include the use of field notes and reflexive journaling throughout the interview and analysis process [59]. After the development of the codebook, each interview will be coded independently by two analysts with discrepancies resolved through discussion; intercoder agreement will be tracked and evaluated using the Cohen’s kappa statistical test. Member checking may be implemented with a subset of participants, although this may be challenging to complete with all participants.

To enhance the quality of the mixed methods meta-inferences we will focus on addressing veracity, consistency, applicability, and neutrality [59]. The quantitative and qualitative quality strategies to increase validity and credibility will contribute to high quality meta-inferences, or overall conclusions of the study. To demonstrate neutrality, we are publishing this protocol paper and will report deviations from the protocol that may occur throughout the study. When reporting the findings, we will provide a clear explanation of how data from each phase contributed to the meta-inference. Quality of the design will be accomplished by using data from the quantitative phase to inform the sample for the qualitative phase, using a maximum variation approach to enhance sample integration [58].

### Integration

In addition to the points of connection between the survey phase and interview phase, the quantitative results and qualitative findings will be mixed to generate meta-inferences or integrated study conclusions regarding factors that contribute to FSA and factors to be addressed through a decisional support intervention. This will involve identifying critical content and resources to include in the decisional support intervention and refining measurement tools and outcomes for the testing of the intervention. Qualitative findings will be integrated with the quantitative descriptive and multivariable model results to further describe decisional satisfaction and associated concepts [61]. This will help to examine decisional satisfaction as a relevant and modifiable intervention outcome and identify factors to be targeted through an intervention.

A joint display will be used to assist with integrative analysis [62]. This will entail creating tables and matrices of quantitative results (e.g., facilitators and barriers to fertility status assessment reported on the survey) and corresponding qualitative themes, sub-themes, and illustrative quotes. The integrated analysis will focus on facilitators and barriers to fertility status assessment that can be incorporated in a decisional support intervention, as well as exploring decisional satisfaction as a relevant intervention outcome. Additional mixed methods integrated analysis approaches could include a linked case analysis, using both quantitative and qualitative data from an individual [61]. Data transformation, likely through quantifying qualitative data, may also be of relevance. It is reasonable to anticipate that qualitative themes related to lack of knowledge about FSA and fertility care could be quantified and then contrasted with overall scores on similar scales from the survey [63].

Qualitative data will be summarized, with illustrative quotes, into matrices by site and FSA status of participant to further identify patterns [64]. Data will be stratified by site to examine whether there are site specific characteristics facilitating or hindering access to FSA. Matrices will be reviewed by the PI and two qualitative analysts; an audit trail will be provided to increase trustworthiness in the findings [65]. These matrices will then be transformed into a descriptive summary of the decision-making process, including influences, facilitators, barriers, and other relevant factors regarding FSA.

### Ethics and dissemination

This study has been approved by the coordinating center institutional review board (Emory University IRB #00003083) and will be approved by all participating site institutional review boards (Cincinnati Children’s, University of Colorado/Children’s Hospital Colorado, Washington University/St Louis Children’s Hospital) prior to recruitment of participants. Informed consent will be obtained from participants prior to completing any aspect of the study. All data will be collected and stored through the password protected and secure REDCap database; audio recordings for qualitative interviews will be stored in password protected files on the secure Emory University server. At each participating site, access to data will be restricted to approved study team members and study-related files will be stored in password protected files. Data will be deidentified whenever possible through the use of assigned study identification numbers. Findings from this study will be disseminated through presentation at academic and professional conferences and in peer-reviewed journal.

## Discussion

Little is known about the perceived reproductive health needs and factors associated with receipt of an FSA among emerging adult female cancer survivors. Overall, prior research has been limited by small sample sizes and lacking in comprehensive assessment of clinical and treatment characteristics, sociodemographic, developmental, and psychological factors related to FSA, all aspects that will be addressed through this study. Through a mixed methods approach, this study will allow for a better understanding of the of factors association with FSA uptake and the decisional processes amongst both survivors who have and have not received an FSA.

This study will use a developmental framework to identify factors related to an FSA among female cancer survivors in emerging adulthood. Studies of reproductive health among cancer survivors have traditionally included a very wide age range (e.g., 15 to 39 years) without accounting for the unique developmental features and milestones that occur during emerging adulthood. There is a lack of developmentally appropriate reproductive health and fertility-related psychoeducational and decisional support interventions available for emerging adult cancer survivors. By focusing on a distinct population of cancer survivors during a developmental period that is ripe for fertility-related distress and family building, results of this study have a strong translational application for survivors who are in their reproductive years. Furthermore, this work will inform future interventions to promote uptake of FSA among interested female cancer survivors.

## Supporting information

S1 File Study protocol. (PDF)
