# Supplementary material for: Examining decisional needs and contextual factors influencing fertility status assessment among young female survivors of childhood cancer: A sequential mixed methods study protocol
Source: PLoS One. 2023 Jun 14;18(6):e0286511. doi: 10.1371/journal.pone.0286511 (PMC10266625; doi:10.1371/journal.pone.0286511)
Supplement: S1 File — (PDF) [file pone.0286511.s001.pdf]

PROTOCOL TITLE: A mixed methods approach to examining decisional needs and contextual factors influencing fertility status assessment outcomes among young female survivors of childhood cancer

PRINCIPAL INVESTIGATOR:

Brooke Cherven, PhD, MPH, RN
Department of Pediatrics, Emory University School of Medicine
Aflac Cancer and Blood Disorders Center at Children's Healthcare of Atlanta
404-727-3117
bcherve@emory.edu

COLLABORATORS

James Klosky, PhD, ABPP
Department of Pediatrics, Emory University School of Medicine
Aflac Cancer and Blood Disorders Center at Children's Healthcare of Atlanta

Ann Mertens, PhD
Department of Pediatrics, Emory University School of Medicine
Aflac Cancer and Blood Disorders Center at Children's Healthcare of Atlanta

Jessica Spencer, MD, MSc
Department of Gynecology and Obstetrics, Emory University School of Medicine
Anne Fitzpatrick, PhD, APRN
Department of Pediatrics, Emory University School of Medicine

Collaborating Site PIs

For each collaborating site, the institution's IRB will review the site PI's engagement in human participants research activities.

Karen Burns, MD, MS
Comprehensive Fertility Care and Preservation Program, Cancer Survivorship Center
Cincinnati Children's Hospital Medical Center
University of Cincinnati College of Medicine

Jenna Sopfe, MD, MS
HOPE Survivorship Program
Children's Hospital Colorado
University of Colorado

Holly Hoefgen, MD
Assistant Professor of Ob/Gyn

Chief, Division of Pediatric and Adolescent Gynecology
Department of Obstetrics and Gynecology
Washington University School of Medicine

STUDY TEAM

Ebonee Harris, BS
Clinical Research Coordinator
Aflac Cancer and Blood Disorders Center
Children's Healthcare of Atlanta

VERSION: 2.0

FUNDING SOURCE: NIH

REVISION HISTORY

Revision #	Version Date	Summary of Changes
1, v 2.0	12-17-21	• Grammar/spelling corrections<br>• Clarified that participating sites will only have access to their own site's data in REDCap<br>• Clarified that the survey is available in Spanish<br>• Provided clarification for recruitment, including the use of Epic My Chart messages, to reflect recruitment procedures across sites<br>• Clarified the maximum number of times a patient may be contacted for recruitment (5 times total, using various methods)<br>• Clarified that the qualitative interviews will be coordinated and conducted by the coordinating center (Emory)<br>• Included the language "Participating sites should follow institutional guidelines regarding internal study monitoring and/or audits." to reflect processes across sites.<br>• A new section titled "Non-participant data" has been added to include the collection of de-identified data for patients who are recruited, but do not participate in the study.<br>• Clarification of the informed consent process, including the option for participating sites to obtain a waiver of documentation of informed consent from their institutional IRB if applicable. The consent processes have been clarified to reflect processes across sites.

Table of Contents

1. Study Summary 5
2. Objectives 6
3. Background 7
4. Study Endpoints 9
5. Study Intervention/Design 9
6. Procedures Involved 10
7. Procedures – Long-Term Follow Up 13
8. Sharing of Results with Participants 13
9. Study Timelines 13
10. Inclusion and Exclusion Criteria 13
11. Population 14
12. Vulnerable Populations 14
13. Local Number of Participants 14
14. Recruitment Methods 15
15. Risk to Participants 16
16. Potential Benefits to Participants 16
17. Compensation to Participants 16
18. Data Analysis, Management and Confidentiality 17
19. Provisions to Protect the Privacy Interest of Participants 19
20. Economic Burden to Participants 20
21. Informed Consent 20
22. Setting 21
23. Resources Available 21
24. Multi-Site Research When Emory is the Lead Site 22
25. References 23

1. Study Summary

**Study Title**	A mixed methods approach to examining decisional needs and contextual factors influencing fertility status assessment outcomes among young female survivors of childhood cancer
**Study Design**	Cross-sectional survey
**Primary Objective**	Explore the perceived reproductive health needs of emerging adult female cancer survivors
**Secondary Objective(s)**	Identify decisional and contextual factors that influence pursuit of fertility status assessment
**Research Intervention(s)/Interactions**	Cross-sectional survey, semi-structured interview
**Study Population**	Female survivors of childhood cancer, aged 18.00-29.99 years with a history of gonadotoxic treatment
**Sample Size**	325
**Study Duration for individual participants**	Survey completion is estimated 20 minutes; semi-structured interviews estimated 45 minutes
**Study Specific Abbreviations/ Definitions**	FSA = fertility status assessment<br>YASCC = young adult survivors of childhood cancer
**Funding Source (if any)**	NIH

2. Objectives

The purpose of this study is to explore the perceived reproductive health needs of emerging adult female cancer survivors and to identify decisional and contextual factors that influence pursuit of fertility status assessment (FSA) using a sequential explanatory mixed method design. This study will inform the development of a future intervention responsive to the specific challenges of female young adult survivors of childhood cancer (YASCC) through a multi-site survey, semi-structured interviews, and an exploration of intervention outcome targets.

Specific Aim 1: Using a quantitative survey of 325 female YASCC (currently aged 18 to 29 years and >1-year post treatment; diagnosed with cancer < age 21 years) determine:

A1.1 Reproductive health knowledge, reproductive values (presence/absence of concerns/distress/desires for parenthood), reproductive health behaviors (pregnancy/birth history, sexual behaviors, contraception use,) and prevalence of reproductive health informational needs

A1.2. Decisional conflict regarding pursuit of an FSA

A1.3. Sociodemographic, developmental, psychological, clinical, and reproductive health factors that are related to receipt of an FSA.

The goal of this aim is to establish a knowledge base of the YASCC perceived reproductive health needs and identify relevant factors related to FSA. This work will inform the development of a future intervention to address FSA decisional needs in female YASCC.

Specific Aim 2: Using a qualitative approach, conduct semi-structured interviews with a subset (n=32) of participants identified through the survey in order to:

A2.1. Describe the contextual factors that influence pursuit of an FSA among female YASCC

A2.2. Identify facilitators and barriers to FSA that can be incorporated into a decisional support intervention

The goal of this aim is to elucidate patient experiences with FSA and preferences for a decisional support intervention.

Specific Aim 3: Identify sociodemographic, developmental, clinical, psychological, and reproductive health factors that are related to decisional satisfaction regarding FSA.
The goal of this aim is to explore decisional satisfaction as an outcome for future interventions and identify factors to be targeted through a decisional support intervention for FSA among female YASCC.

3. Background

Childhood cancer survivor population: characteristics and health risks. Cancer treatment has improved such that survival rates overall for childhood and adolescent cancer now surpass 80%.[1] There are nearly 400,000 survivors of childhood/adolescent cancers in the United States, most of whom are now young adults and in their reproductive years. Survivors who received gonadotoxic therapies (e.g. alkylating chemotherapy, radiation affecting the gonads, hematopoietic stem cell transplant, and/or surgery impacting the gonads) are at risk for cancer treatment-related infertility.[2] Infertility rates range from 11-26% among young adult female cancer survivors[3] and when compared with healthy siblings, female survivors are more likely to experience infertility[4] and less likely to report a pregnancy[5]. A large proportion of female survivors of childhood cancer receive gonadotoxic treatment – representing a population who may be at risk for a shortened window of fertility – and female cancer survivors may be eligible for fertility preservation options post cancer treatment.[6-8]

Cancer survivors overwhelmingly report a desire for children. Future fertility is a priority among patients and families from the time of cancer diagnosis[9] into long-term survivorship[10-14]. Across multiple studies of female survivors of childhood cancer, >75% report a desire for children in the future.[11,15,16] Fertility preservation options prior to cancer treatment are limited for adolescent females (e.g., oocyte cryopreservation) due to urgency of treatment initiation. Until recently, the only option for prepubertal females – ovarian tissue cryopreservation – was considered experimental.[17] While initiatives are underway to increase access to fertility preservation options at the time of cancer diagnosis, only a small minority of female cancer survivors have cryopreserved oocytes or ovarian tissue.[18-20] After cancer treatment, potential infertility is a substantial source of distress for young adult cancer survivors[21,22] and has been associated with depression, anxiety, stress, and trauma[21,23-25]. The overall prevalence of fertility-related distress or worry ranges from 20-60% among young adult survivors of cancer and, compared with males, females reported higher distress.[21,26]

Despite concerns, cancer survivors are uncertain of their fertility status. After gonadotoxic treatment, patients desire information regarding their risk for infertility[27] and providing tailored education to survivors is successful in improving accurate perceptions of infertility risk[28]. Even with an accurate perception of risk for infertility, many survivors are unaware of their fertility status.[29] Uncertain fertility status is reported by 48-77% of survivors of childhood cancer, despite their interest in biological children, underscoring the need for interventions to increase uptake of fertility status assessment among interested survivors.[26,30-32] Over half of 179 female cancer survivors (mean age 29 years) report unmet information needs regarding their options to assess and preserve fertility during survivorship, and unmet needs were associated with greater decisional

conflict regarding fertility preservation post cancer treatment, while the receipt of a fertility evaluation was related to lower decisional conflict.[33] In qualitative studies of young adult survivors, females describe the psychological burden of uncertain fertility, pressure on their family building timeline due to possible premature menopause, and missing out on the shared peer and social experiences associated with pregnancy.[34,35]

Cancer survivors are interested in fertility status assessment. A fertility status assessment (FSA) for females can include medical history, assessment of menstrual-cycle specific laboratory hormones and antral follicle count, and discussion of ovarian reserve and fertility potential[36], generally through a consult with a fertility specialist (e.g., reproductive endocrinologist). While assessment of gonadal function is sometimes completed in a cancer survivor clinic, these tests are not definitive, and estimation of future fertility can be difficult. The gold standard for fertility assessment is an antral follicle count[36], which is best completed by a reproductive specialist who can provide a perspective beyond that of treatment-related risk. Among survivors at risk for infertility, FSA is recommended in accordance with national guidelines among interested patients.[37] In a sample of 98 young adult cancer survivors, 71% of females reported interest in a FSA and over half were interested in further reproductive health information delivered via a website or follow-up visit.[28] Females who are at risk for premature ovarian insufficiency may have a window for fertility preservation, therefore timely FSA provides an opportunity for discussing options for fertility preservation, and potentially intervening in a timely fashion in order to improve preservation outcomes. However, little is known regarding factors related to receipt of an FSA in female survivors of childhood cancer.

Factors related to receipt of an FSA. Survivors of childhood cancer who have pursued FSA describe a desire to be prepared for the future, while others have not pursued assessment because they are worried about the finality of the results if they are infertile.[38] Among 45 survivors aged 22-44 years who had not undergone and FSA, the top barrier was being unaware of the option for FSA (44%), followed by cost (24%), lack of access to reproductive services (22%), postponing FSA until in a relationship (20%) or until they are older (17%), while 15% did not desire children and 13% reported they would be afraid of the results.[30] In a qualitative study of adult cancer survivors, females describe a second wave of trauma after cancer when dealing with potential infertility, exacerbated by direct and indirect financial concerns and toxicity (e.g., cancer and infertility-related medical costs, stalled job growth, mounting debt).[39] Financial concerns are also a substantial source of distress among adult survivors pursuing family-building (i.e., costs related to assisted reproductive technology, surrogacy, adoption), and may impact the decision to pursue FSA.

Interventions to address unmet fertility-related needs must include a developmental approach. Emerging adulthood represents a developmental period of transition from adolescence to adulthood. Emerging adults are diverse in their level of

independence and achievement of milestones, including educational attainment, employment, and relationship status, which may impact a survivor's pursuit of an FSA.[40] Psychologically, emerging adulthood is a time of experiencing possibilities, exploration, and feeling 'in between' childhood and adulthood.[40] During emerging adulthood, cancer survivors may be newly accessing reproductive health services, forming committed romantic relationships, and navigating the expectations of friends and family members regarding family-building. In this developmental context, emerging adulthood may be a time when survivors are anxious to explore their options for biological parenthood through an FSA.

Preliminary Data Retrospective medical record data from cancer survivors in the Aflac Cancer Survivor Clinic at Children's Healthcare of Atlanta were collected. Preliminary analyses of 77 female cancer survivors (mean age 19.2±1.5 years; range 18-25 years) demonstrate that 57.1% of survivors reported worry about future infertility during the clinical encounter, 50.6% were interested in pursuing FSA at some point in the future, and 13% had documented prior receipt of an FSA. Univariable logistic regression demonstrated that female YASCC who endorsed worry about infertility were more likely to indicate an interest in FSA (Odds Ratio [OR] 15.9, 95% Confidence Interval [CI] 3.7-89.1; $p<0.001$). These preliminary data suggest that a substantial proportion of female YASCC in emerging adulthood are worried about infertility and interested in an FSA, and that comprehensive assessment of self-reported factors that influence pursuit of an FSA is warranted.

4. Study Endpoints

The first study endpoint is the completion of quantitative data collection, through web-based survey. The second study endpoint is the completion of qualitative data collection, through semi-structured interviews. This study is minimal risk and therefore does not include safety endpoints.

5. Study Intervention/Design

This study will utilize an explanatory mixed methods approach to elucidate factors that are related to receipt of an FSA among emerging adult (18-29 years of age) female cancer survivors. Employing a mixed methods approach will allow for a comprehensive exploration of the experiences of young female cancer survivors and their decision to pursue FSA.

Figure 1. Study conceptual model

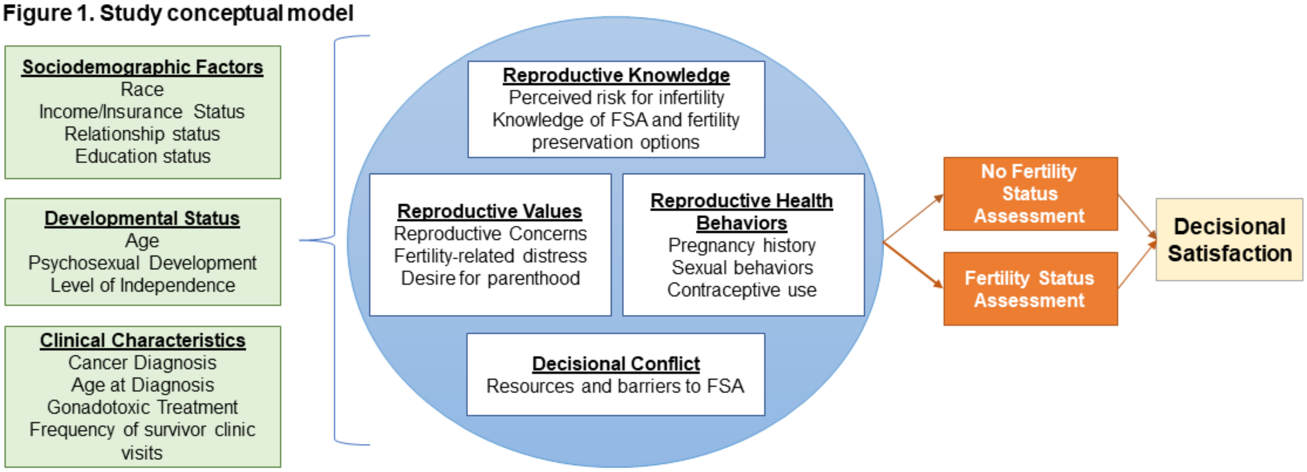

The conceptual model for this study (Figure 1) is informed by the Ottawa Decision Support Framework that purports an individual's decisional needs will affect decision quality, behavior, and emotions regarding the decision.[41,42] This framework has been used to develop successful decisional aids for fertility preservation at the time of cancer diagnosis[43,44], and in this study will guide an assessment of decisional needs for females who have completed cancer treatment. Decisional needs can include an individuals' sociodemographic and clinical characteristics, knowledge and values regarding the decision, and resources to make or carry out the decision. Decisional conflict – a state of uncertainty about an action (e.g., pursuit of an FSA) – is associated with decisions that have uncertain outcomes and potential gains and losses, which can result in delayed decision making.[41,45] Decisional support interventions address these factors, resulting in an informed and values-based decision. This study will include a comprehensive assessment of decisional needs as they relate to pursuit of an FSA among female cancer survivors; these data will inform the development of a future decisional support intervention.

In this study we propose that sociodemographic and developmental factors, along with cancer clinical characteristics, influence reproductive knowledge, values, and behaviors. These factors inform the individual's values regarding fertility and desire for FSA. In this context, cancer survivors may experience decisional conflict regarding FSA; decisional satisfaction will be explored as an outcome of the decision to pursue/not pursue FSA.

6. Procedures Involved

Study participants will complete a web-based survey reporting reproductive health needs, fertility-related outcomes, and pursuit of a fertility status assessment. Clinical characteristics will be abstracted from participant's medical records. Both the survey data and clinical data will be entered and stored in Children's Healthcare of Atlanta REDCap (http://project-redcap.org/), which is a secure, web-based application for building and managing online surveys and databases. Participating sites will only have access to their own site's data; the coordinating center (Emory/CHOA) will have access to all data across sites. REDCap is HIPAA compliant and designed to support data capture for research studies, providing an intuitive interface for validated data entry;

audit trails for tracking data manipulation and export procedures; automated export procedures for seamless data downloads to statistical software packages; and procedures for importing data from external sources A subset of participants will be invited to participate in a qualitative interview. Interviews will be audio-recorded and transcribed verbatim. Audio recordings will be stored in a password-protected secure folder on the Emory server and destroyed after data analysis is complete. Figure 2. Presents the mixed methods procedural diagram for this study.

Figure 2. Mixed methods procedural diagram

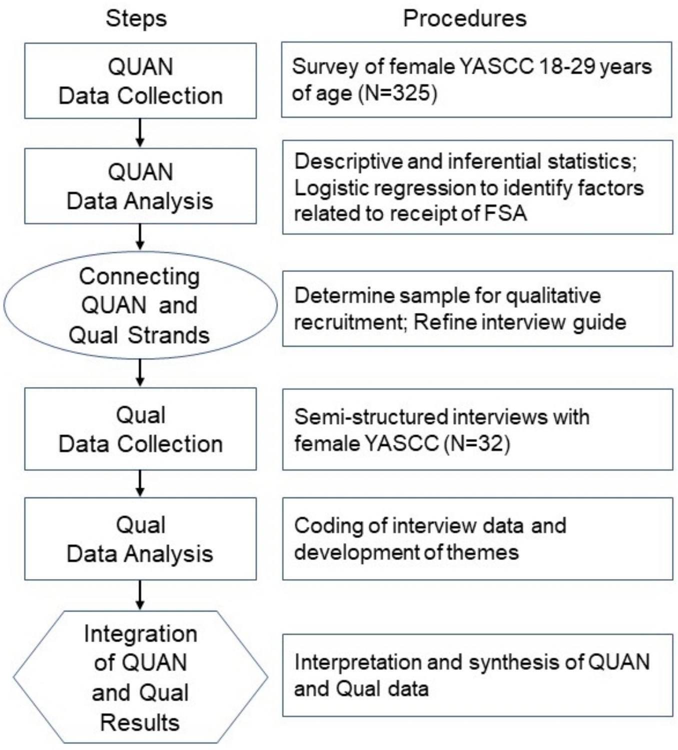

<u>Data to be collected</u>

<u>Medical Record Data</u>: **Clinical Characteristics**, including cancer diagnosis, date of diagnosis, number of cancer survivor clinical[41] encounters, hormonal laboratory assessments, prior hormone treatment, gonadotoxic therapeutic exposures [alkylating and heavy metal chemotherapy, gonadoxic radiation], surgeries, history of hematopoietic cell transplant, and date of cancer treatment completion will be abstracted from participant's medical records by the site CRC using an adapted version of the COG Summary of Cancer Treatment Template.[46]

<u>Quantitative Survey Data</u>: Participants will be invited to complete a web-based REDCap survey assessing sociodemographic and developmental factors, reproductive factors (knowledge, concerns, behaviors, and health needs), psychological factors, and history of FSA. The survey instrument is included with this protocol.

Sociodemographic factors include gender identity, race/ethnicity, religion, education level, household income, insurance and relationship status, and items are consistent with national surveys of young adults.[47] **Developmental factors** will be assessed through the lens of emerging adulthood using The Markers of Adulthood (MoA)[48,49] scale, which characterizes the attainment of behavioral milestones among emerging adults, and the IDEA-8[50] characterizes a self-perceived state of transition (e.g., feeling in-between); both scales have been used in large multi-site studies with emerging adults across diverse populations.[51]

Reproductive knowledge will be assessed using an overall score of correct answers (0-12) to items regarding cancer and fertility preservation.[52] Gaps in knowledge will be assessed through self-reported knowledge level (item response options include none, a little, a lot) regarding fertility after cancer (e.g., impact of cancer treatment on fertility, knowledge of infertility testing and treatment).[53,54] Survivors will also report their perceived risk for infertility (none, low, moderate, high) and how they perceive their risk for infertility compared with peers who have not had cancer.[29] The modified **Reproductive Concerns Scale (mRCS)** consists of 7 items with 3 subscales measuring participant's fertility-related concerns, illness concerns related to pregnancy/parenthood, and information-seeking regarding reproductive health. The mRCS has been validated among adolescent and young adult survivors of childhood cancer.[55,56] **Fertility-related distress** will be assessed using three domains of the Fertility Problem Inventory (FPI)[57,58], a 27-item multidimensional scale measuring infertility stress, impact on social dimensions, and importance of parenthood and the Couple's Relationship Concern subscale from the Reproductive Concerns After Cancer scale[59,60]; higher scores indicate increased distress. **Reproductive health behaviors** will include pregnancy and birth history, contraceptive history and current use, and sexual behaviors.[47,61] **Reproductive health needs** will be assessed through items identifying unmet educational needs, desire for contraceptive counseling, and interest in FSA.

Psychological factors will also be assessed. **Decisional conflict** will be measured using the Decisional Conflict Scale, which measures four dimensions of decision making: information, values, support, and uncertainty; each subscale is scored 0-100 with higher scores indicating greater decisional conflict. The effective decision subscale will be used to measure **decisional satisfaction** among participants who have received an FSA; scores range from 0-100 with lower scores representing a good decision.[45]

Fertility Status Assessment will be assessed with the item "*Have you ever met with a reproductive specialist to assess your fertility? (e.g. reproductive endocrinologist or a fertility specialist)*". Participants who respond 'Yes' will also be asked details about this assessment (e.g., testing, results, discussion of infertility treatment options). Participants who respond 'No' or 'I don't know' will be asked their level of interest (Likert scale 1 [not at all interested] to 5 [very interested]), and perceived barriers to not receiving an FSA (e.g., lack of awareness, cost).[30]

Qualitative Interview Data: Participants for the qualitative interviews will be identified through analysis of the quantitative survey if they (1) indicated on the survey that they are willing to take part in a qualitative interview and (2) responded to the survey item assessing receipt of an FSA. The interviewers for this study are English-speaking, therefore only participants who speak English will be invited to take part in the qualitative interview. We will use a qualitative descriptive approach, which is low-inference and seeks to describe the experiences of participants through their words and

experiences.[62] In line with this approach, we will use maximum variation sampling, which is a preferred method for descriptive qualitative inquiry, to increase the representativeness of the sample in relation to the population being studied by recruiting participants across sites and those who have and have not received an FSA.[63,64]

Interviews will be conducted until data saturation is reached; we estimate the sample size to be 32 participants, (8 at each participating site). We will try to have approximately equal number of participants who have and have not received FSA at each site, however, some sites may have higher success rates testing patients, therefore stratification of patients by FSA status may not be possible at the site level. However, we will ensure overall there are approximately 16 participants who have received an FSA and 16 who have not received an FSA.

The interview guide will be developed and informed by descriptive results from the quantitative survey. Planned interviews questions include a discussion of factors that influenced participants' decision to pursue or not pursue FSA, satisfaction with that decision, barriers encountered when accessing FSA, and resources needed to support survivors who are interested in FSA.

7. Procedures – Long-Term Follow Up

There are no long-term follow-up procedures.

8. Sharing of Results with Participants

Study results will not be shared with participants or others, beyond scholarly dissemination of de-identified data through presentations and publications.

9. Study Timelines

The estimated time for participants to complete the quantitative survey is 15-20 minutes; for participants who complete a qualitative interview, the estimated time is 45 minutes. We anticipate active study recruitment and data collection to be complete within 3 years.

10. Inclusion and Exclusion Criteria

The population of interest for this study is female survivors of childhood cancer, 18 through 29 years of age.

Inclusion Criteria:
(1) 18.00 to 29.99 years of age
(2) diagnosis of cancer < 21.00 years of age
(3) female sex
(4) received gonadotoxic cancer treatment (e.g., alkylating chemotherapy agents, heavy metal chemotherapy, radiation including the gonads, cranial radiation ≥ 30 Gy, or hematopoietic cell transplant)
(5) >1 year from completion of cancer treatment

Exclusion Criteria:
(1) cognitive dysfunction, such that they would be unable to complete the survey
(2) bilateral oophorectomy

11. Population

The purpose of this study is to explore reproductive health and identify factors related to receipt of a fertility status assessment among female young adult cancer survivors. This study will enroll 325 female cancer survivors aged 18.00 through 29.99 years.

Pregnant women will not be excluded from participating in this research. Adults unable to consent, those with cognitive dysfunction, or individuals with impaired decision-making capacity will be excluded as they are unable to complete the study measures. Individuals who speak English or Spanish will be included in the quantitative survey aim of this study, which will be available in English and Spanish; only English-speaking individuals will be eligible to participate in the qualitative interviews.

12. Vulnerable Populations

It is possible that some participants may be pregnant. We do not anticipate any additional risks from completing the survey or qualitative interview for participants who are pregnant. Strategies to ensure privacy, confidentiality, and reduce discomfort will be the same as for participants who are not pregnant.

Children will not be enrolled in this study. The health needs and decisional factors related to fertility assessment are influenced by age and development, therefore this study will be limited to focus on the unique developmental period of emerging adulthood. The minimum age limit (18.00 years) was chosen to include participants who are legally able to make independent medical decisions, such as pursuing a fertility status assessment.

13. Local Number of Participants

The study will recruit 325 participants study-wide, with 100 participants enrolled locally at the Children's Healthcare of Atlanta/Emory University site. Participant eligibility will be confirmed prior to recruitment.

The purpose of this study is to explore reproductive health and decisional factors regarding fertility status assessment. The sense of urgency and factors that influence the decision to pursue a fertility status assessment are different for females and males,

therefore our planned enrollment includes 100% female participants. Transgender men will also be eligible for this study; gender identity will be self-reported as part of the survey data collection.

Participants of any race and ethnicity will be recruited for this study. Estimates of race and ethnicity enrollments are derived from site-specific databases that were used to estimate demographics of survivors who would meet the eligibility criteria for this study. The race and ethnicity of survivors vary across sites and are related to the demographic characteristics of the geographical region. Overall, across all sites, we anticipate 76.9% of participants will be white, 12.6% Black, 6.4% more than one race, 2.2% Asian, 1.2% American Indian/Alaska Native, 0.6% Native Hawaiian or other Pacific Islander. We estimate that 13.1% of white participants will be Hispanic and 2.4% of Black participants will be Hispanic. These estimates align with previously published population-based studies of survivors of childhood cancer. The study survey will be available in Spanish for completion on REDCap or via paper and pen.

14. Recruitment Methods

Recruitment procedures are based on strategies that have been successful with our research team in the past.[27,65] The PI and research coordinator at each site will use site-specific databases, cancer registry data, or similar methods to identify patients who meet eligibility criteria (i.e., female sex, aged 18 through 29 years, history of cancer diagnosis <21 years of age, > 1 year from completion of cancer treatment, history of gonadotoxic treatment exposures). This approach has already been established and utilized at each site in order to provide details on estimated eligible participants. Once the list of potentially eligible patients is finalized, the list will be stratified by age group (18.00-24.99 and 25.00-29.99). Then each site will randomize their list, using excel or similar software, to begin consecutive recruitment. This approach will contribute to a more representative study sample. Site study teams will examine the medical records for patients prior to recruitment to verify eligibility.

The site research coordinator will recruit patients using various methods, depending on institutional approvals which include:

Email/Epic MyChart. An email/MyChart invitation including a link to the REDCap survey is the primary and preferred method of recruitment. The email will explain the purpose of the study, briefly outline the study procedures, and include site-specific contact information; there will also be instructions for patients to indicate if they decline participation and do not wish to be contacted further. Patients who are interested will click on the survey link. Participants will complete the REDCap survey (estimated time 15-20 minutes) and following completion the participant will be asked to provide contact information to receive the study gift card. A reminder email(s)/My Chart message can be sent for patients who do not respond.

Phone Call: For patients who do not complete the survey after receiving reminder emails/My Chart message, patients for whom email addresses are not available, and

sites who are unable to use email recruitment, will contact patients by phone. The site research coordinator will describe the purpose of the study and patients who are interested will be emailed a link to the REDCap survey or will be provided with a web address to access the survey. Patients who are unable or choose not to complete the survey online will be mailed a paper copy, with a self-addressed stamped envelope to return to the coordinating site. The study research coordinator will enter participant responses from paper surveys into the REDCap database.

Mail: Sites may recruit participants by mailing a letter describing the study and inviting participation.

In-person: Finally, if eligible patients have scheduled clinical visits within the oncology center, the research coordinator may recruit patients during the clinical encounter.

Recruitment methods will be tracked by the site research coordinators. Recruitment approaches may vary across sites to align with institutional processes. Patients may be contacted up to 5 times total for recruitment, which may be a combination of email/My Chart, phone call, or letter.

Qualitative Interview Recruitment

Participants will indicate on the REDCap survey if they are willing to take part in a qualitative interview and will provide contact information for scheduling. Participants will be contacted by the coordinating center research coordinator and invited to take part in the interview. Interviews will be scheduled at a time that is convenient for the participant and will be conducted remotely via phone call or Zoom (HIPAA compliant platform available through Children's Healthcare of Atlanta). The qualitative interviews will be coordinated and conducted by the study coordinating center (Emory/CHOA).

15. Withdrawal of Participants

There are no anticipated circumstances under which participants will be withdrawn from the research without their consent. Participants can choose to stop participating in the study at any time by contacting the research coordinator or PI to sign a "Request for Withdrawal of Authorization".

16. Risk to Participants

This study poses minimal risks to participants. It is possible that participants may become uncomfortable or upset when answering survey items about reproductive health and potential infertility. Similarly, participants may experience emotional discomfort when discussing infertility and/or prior cancer treatment during qualitative interviews. Participants will be able to skip survey questions, take a break during the interview, and stop the survey and/or interview at any time if they become uncomfortable or upset. Contact information for their cancer survivor program will be provided if they have questions or wish to follow-up with a healthcare provider. There is

also a risk for breach of confidentiality for participants. A breach of confidentiality could negatively impact a research participant who would not want their health information, or their participation in a research study, shared with those outside of the research team.

Participating sites should follow institutional guidelines regarding internal study monitoring and/or audits.

17. Potential Benefits to Participants

Participants may not experience a direct benefit from participating in this study. It is possible that participants may benefit from reflecting on their values regarding future fertility through items on the survey or from discussing potential infertility during the interviews. The information gained from the survey and interviews will inform future interventions focused on reproductive health among young female cancer survivors.

<u>Importance of the knowledge to be gained</u>

Potential infertility is a source of distress among young adult cancer survivors. Interventions supporting female cancer survivors who are at risk for treatment-related infertility and fertility-related distress are a priority. Knowledge gained from this mixed methods study will inform the testing of a decisional support intervention and may benefit cancer survivors in the future. Results from this study can improve clinical care of cancer survivors through the development of appropriate reproductive health resources and counseling.

18. Compensation to Participants

After completing the quantitative survey, participants will receive a $10 gift card. Participants who complete the qualitative interview will receive a $30 gift card. Gift cards will be sent by the coordinating center within approximately one month after completion of the survey and interview (if applicable).

19. Data Analysis, Management and Confidentiality

<u>Aim 1. Data Analysis</u>: Statistical analysis will be performed using SAS v.9.4 (Cary, NC) and CRAN R v.3.6 (Vienna, Austria), and statistical significance will be evaluated at the 0.05 threshold. Initial analyses will be undertaken to inspect data for errors, inconsistencies, and incomplete information. Data anomalies and outliers will be examined and corrected or removed if necessary. Descriptive statistics for sociodemographic and developmental factors, and clinical characteristics will be presented as means and standard deviations or medians and interquartile ranges for continuous variables and frequencies/percentages for categorical variables.

The <u>primary outcome of Aim 1</u> is receipt of an FSA, defined as an answer of ‘yes’ to the survey item *“Have you ever met with a reproductive specialist to assess your fertility?”.* Based on prior research, and clinical experiences at the participating sites, we anticipate 20% of the sample will report receipt of an FSA.[12,30,66]

Aim 1.1. and **Aim 1.2**: Descriptive statistics for reproductive health knowledge, values, health behaviors, and prevalence of reproductive health needs, as well as decisional conflict and fertility-related psychological distress, will be examined using means and standard deviations, medians and interquartile ranges, or percentages and frequencies, as appropriate. For the primary outcome variable (i.e., “yes” for an FSA), frequency and percentage will be calculated with a 95% confidence interval. Hypothesis tests between FSA cohorts (i.e., yes versus no) will be made using two-sample t-tests and chi-square tests of independence (or non-parametric equivalents such as Wilcoxon rank-sum and Fisher’s exact tests); moreover, effect sizes will be calculated to determine standardized differences in exposure values between the FSA cohorts and interpreted using Cohen’s *d* criteria: small (0.2), moderate (0.5), and large (0.8).

Aim 1.3: To assess associations between model parameters describing sociodemographic, clinical, reproductive health needs and decisional conflict (covariates) and receipt of an FSA (outcome), we will employ bivariable and multivariable binary logistic regression models. For all logistic regression models, model parameters for sociodemographic, clinical, reproductive health needs and decisional conflict will be treated as fixed effects and study sites will be evaluated as random effects, where appropriate, to account for potential differences in outcome variability between the study locations (i.e., clustering); moreover, fit statistics will be gauged (i.e., AIC, BIC, log-likelihood) for all regressions and appropriate assumptions will be checked, such as linearity in covariates and normality of residuals. Final multivariable results will be guided by the bivariable associations and determined using backward selection procedures, as well as any relevant clinician input. Estimates from these models will be presented as odds ratios for receipt of an FSA with 95% confidence intervals and p-values.

Power Analysis: The primary endpoint for this study will be receipt of an FSA. Based on insights from the literature, and our own clinical experiences, we anticipate 20% (n=65) of the sample will have received an FSA and 260 respondents will have not. These sample sizes, which will be obtained by sampling 4 study sites, achieve 80% statistical power to detect an odds ratio of 2.4 for discrete exposures. This odds ratio corresponds to 70.4% of respondents in the FSA group having the exposure, versus 50% of respondents in the non-FSA group having the exposure (i.e., a percent difference of 20.4%). Power was calculated in PASS v.14.0.8 (Kaysville, UT), with a two-sided un-pooled Z-test, an intra-cluster correlation (ICC) of 0.01, and a statistical significance level of 0.05.

Non-participant data

In order to evaluate the representativeness of the sample enrolled in this study, we will compare participant and non-participant demographics and clinical characteristics. The data will include age, race/ethnicity, and cancer diagnosis category (leukemia,

lymphoma, solid tumor). Sites will report de-identified data for eligible patients who were recruited, but either declined or were unable to be contacted.

<u>Aim 2. Data Analysis</u>: Qualitative analysis will be supported by experts in the Emory Intervention Development, Dissemination, and Implementation (IDDI) research group. In-depth interviews will be audio-recorded and professionally transcribed verbatim by members of the IDDI group and checked for accuracy. NVivo (v12), will be used to manage and analyze all qualitative data. The codebook will be developed based on the interview guide and emergent themes identified through open coding of the first few transcripts and team discussions.[67] After the development of the codebook, each interview will be coded independently by two analysts with discrepancies resolved through discussion; intercoder agreement will be tracked and evaluated using the Cohen's kappa statistical test. We will generate node reports (e.g., text associated with a specific code) using NVivo to facilitate identification of sub-themes and similarities and differences by group (i.e., site and receipt of FSA, as well as sociodemographic factors). Patient data will be summarized, with illustrative quotes, into matrices by site and FSA status of participant to further identify patterns.[68] Data will be stratified by site to examine whether there are site specific characteristics facilitating or hindering access to FSA. Matrices will be reviewed by the PI and two qualitative analysts; an audit trail will be provided to increase trustworthiness in the findings.[69] These matrices will then be transformed into a descriptive summary of the decision-making process, including influences, facilitators, barriers, and other relevant factors regarding FSA. IDDI, led by Dr. Michelle Kegler who has extensive qualitative expertise, will conduct the qualitative analyses in conjunction with the PI.

A2.2 The quantitative results and qualitative findings will be combined to generate meta-inferences regarding factors that contribute to FSA and barriers to be addressed through a decisional support intervention. This will include identifying critical content and resources to include in the decisional support intervention and refining measurement tools and outcomes for the testing of the intervention.

<u>Aim 3. Data Analysis</u>: To assess associations between model parameters describing sociodemographic, developmental, clinical, psychological, and reproductive health factors (covariates) and decisional satisfaction regarding FSA (outcome), we will employ bivariable and multivariable general linear regression models. Decisional satisfaction scores range from 0-100, with lower scores representing a good decision. Similar to methods for Aim 1.3, model parameters for sociodemographic, clinical, reproductive health needs and decisional conflict will be treated as fixed effects and study sites will be evaluated as random effects, as appropriate. Estimates from the regression models will be presented as slopes and least-squares means with 95% confidence intervals, p-values and effect sizes. Data transformations, including natural logarithms or inverse hyperbolic sine, may be applied to meet model assumptions, and if normality cannot be achieved, non-parametric methods or alternative modeling strategies (such as generalized additive models or regression splines) will be employed. Final multivariable

results will be guided by the bivariable associations and determined using backward selection procedures and any relevant clinician input.

Qualitative data will be integrated with the descriptive data and multivariable model results to further describe decisional satisfaction and associated concepts. This will help to examine decisional satisfaction as a relevant and modifiable intervention outcome and identify factors to be targeted through an intervention.

Data Management

All data will be collected and stored through the password protected and secure REDCap database; audio recordings for qualitative interviews will be stored in password protected files on the secure Emory University server. At each participating site, access to data will be restricted to approved study team members and study-related files will be stored in password protected files. If a participant completes the survey using paper and pencil, the hard copy of the survey will be stored at the participating site in a locked drawer and data will be entered into REDCap by the approved site CRC.

Data will be coded whenever possible through the use of assigned study identification numbers. Upon dissemination of results, no identifiable data will be included in presentations or publications. All study team members will maintain certification in human subjects research through the Collaborative Institutional Training Initiative (CITI) program. To protect privacy, participants will be encouraged to complete the web-based REDCap survey in a private area. Similarly, qualitative interviews will be scheduled during a time when participants can have privacy.

20. Provisions to Protect the Privacy Interest of Participants

In order to ensure privacy and confidentiality of data several strategies will be employed. All data will be collected and stored through the password protected and secure REDCap database; audio recordings for qualitative interviews will be stored in password protected files on the secure Emory University server and audio recordings will be destroyed after data analysis is complete. At each participating site, access to data will be restricted to approved study team members and study-related files will be stored in password protected files. Data will be deidentified whenever possible through the use of assigned study identification numbers. Upon dissemination of results, no identifiable data will be included in presentations or publications. All study team members will maintain certification in human subjects research through the Collaborative Institutional Training Initiative (CITI) program. To protect privacy, participants will be encouraged to complete the web-based REDCap survey in a private area. Similarly, qualitative interviews will be scheduled during a time when participants can have privacy.

Quantitative Survey The voluntary nature of this study will be explained during recruitment and through the informed consent document. If a participant becomes

uncomfortable or distressed when completing the survey, they will be able to stop participation or skip items that they do not wish to answer. All participants will be provided with contact information for their local cancer survivorship clinic and reproductive specialist, if they have medical questions or wish to learn more about their potential for treatment-related infertility.

Qualitative Interviews Participants will only be invited to take part in the qualitative interview if they indicated interest on the survey. Interviews will be conducted by experienced interviewers, trained in the study protocol and who have experience with discussions of sensitive health information. If a participant becomes distressed, they will have the opportunity to take a break or end the interview at any point.

21. Economic Burden to Participants

There are no anticipated costs associated with participation in this study.

22. Informed Consent

Participating sites will follow institutional guidelines regarding consent processes. This may include a waiver of documentation of consent (and providing the participant with an informational sheet about the study as part of the recruitment process). Some sites may use e-consenting procedures, verbal consent, or written consent (details below).
Each site will obtain institutional IRB approval.

E-consenting:
The preferred participant contact method will be via email where they will be sent a link to REDCap where they will sign an e-consent.

The initial page of the REDCap survey will contain a brief written consent outlining the purpose of the study, its voluntary nature, time commitment, incentive information, and release of PHI, the names and contact information for the study PI and the appropriate Institutional Review Board will also be included. To record participants' consent to participate, we will have them click a radio button labeled "Yes, I agree to take part" or "No, I do not want to take part". We will also have participants insert the current date and their name. Participants who select "Yes, I agree to take part" will be routed to the study survey battery. Participants who select "No, I do not want to take part" will exit the REDCap system and route to a screen that thanks them for their consideration.

Verbal consent may be obtained from participants over the phone, using IRB approved language.

Participants recruited in person may be consented with a written IRB approved form. Hard copies of the informed consent document will be available for their review and signature; these documents will be stored in a secure location at each study site. The survey will be provided to them immediately following their consent discussion.

Sites will follow institutional guidelines for obtaining consent from Spanish-speaking participants.

23. Setting

Potential participants will be identified and recruited from the Aflac Cancer and Blood Disorders Center at Children's Healthcare of Atlanta/Emory University, Atlanta, GA and three external participating sites:

(1) Cincinnati Children's/University of Cincinnati, Cincinnati, OH
(2) Children's Hospital Colorado/University of Colorado, Aurora, CO
(3) St Louis Children's Hospital/Washington University, St Louis, MO

At each participating site, eligible survivors will be identified using institutional databases, cancer registries, and/or the electronic medical record. Participants will be recruited through email/My Chart, phone call, mail, or in-person and invited to complete the web-based survey. Participants may choose to complete the survey via pencil and paper. Qualitative interviews will take place over the phone or via Zoom and will be conducted by trained personnel from the coordinating center (Emory University).

The institutional review board at each participating site will review and approve the study prior to any site study activities.

24. Resources Available

Recruitment will be stratified across age groups, with emphasis on the early years of emerging adulthood to ensure a developmentally-diverse sample; we will recruit two-thirds of the sample from participants 18.00-24.99 years of age (n=215) and one-third from participants 25.00-29.99 years of age (n=110). The estimated number of eligible patients by participating site is presented in **Table 1**. The pool of potential participants at each site is quite large, with an estimated 1758 female cancer survivors that meet eligibility criteria across sites. These estimates were obtained using site-specific clinical databases and cancer registry data. The enrollment target of 325 is just under 20% of those estimated to be eligible.

Table 1. Anticipated recruitment by participating site and age

	Estimated eligible by age stratification		Total estimated eligible	Planned recruitment
Participating Site	18.00-24.99y	25.00-29.99y		
Emory	376	105	481	100
U of Cincinnati	297	148	445	100
U of Colorado	355	177	532	75
WashU/STL	201	99	300	50
**Total**	**1229**	**529**	**1758**	**325**

This study will recruit survivors from Emory University/Children's Healthcare of Atlanta, and three collaborating institutions, the University of Cincinnati/Cincinnati Children's, University of Colorado/Children's Hospital Colorado, and Washington University/St. Louis Children's Hospital. At each site, the site PI will oversee all aspects of the study, including maintaining regulatory and Institutional Review Board records, the identification and recruitment of eligible participants, and oversight of the site research coordinator. Sites will identify eligible patients using institutional databases or cancer

registries. The PI and site research coordinator will invite eligible patients to participate through email, mail, phone call, or during a routine in-person clinical visit.

25. Multi-Site Research When Emory is the Lead Site

This study will recruit eligible participants from three external sites. Collaborative site research teams will consist of a site PI and clinical research coordinator. The site PI will be responsible for obtaining site IRB approval, identifying eligible participants at their site, supervising the site research coordinator, and overseeing site activities. The clinical research coordinator will be responsible for recruiting participants, completing the Treatment Summary form for participants, and communicating with the coordinating center.

The PI will monitor recruitment across sites through several strategies. Regular communication will occur between the study PI and the site PIs to discuss any challenges in identifying or recruiting eligible patients. The site study teams will complete a bi-weekly recruitment report detailing progress and noting any problems with recruitment. If needed, changes will be made to the recruitment process to meet the needs of each participating site (e.g., relying more heavily on phone or mail recruitment if email recruitment is not successful).

26. References

1. Howlader N, Noone, AM., Krapcho, M., Miller, D., Brest, A., Yu, M., Ruhl, J., Tatalovich, Z., Mariotto, A., Lewis, DR., Chen, HS., Feuer, EJ., Cronin, KA.,: SEER Cancer Statistics Review, 1975-2016, National Cancer Institute. Bethesda, MD, National Cancer Institute, 2019
2. Hudson MM, Ness KK, Gurney JG, et al: Clinical ascertainment of health outcomes among adults treated for childhood cancer. Jama 309:2371-2381, 2013
3. Chemaitilly W, Cohen LE: DIAGNOSIS OF ENDOCRINE DISEASE: Endocrine late-effects of childhood cancer and its treatments. Eur J Endocrinol 176:R183-r203, 2017
4. Barton SE, Najita JS, Ginsburg ES, et al: Infertility, infertility treatment, and achievement of pregnancy in female survivors of childhood cancer: a report from the Childhood Cancer Survivor Study cohort. Lancet Oncol 14:873-81, 2013
5. Green DM, Kawashima T, Stovall M, et al: Fertility of female survivors of childhood cancer: a report from the childhood cancer survivor study. J Clin Oncol 27:2677-85, 2009
6. Poorvu PD, Frazier AL, Feraco AM, et al: Cancer Treatment-Related Infertility: A Critical Review of the Evidence. JNCI Cancer Spectr 3:pkz008, 2019
7. Chemaitilly W, Li Z, Krasin MJ, et al: Premature Ovarian Insufficiency in Childhood Cancer Survivors: A Report From the St. Jude Lifetime Cohort. J Clin Endocrinol Metab 102:2242-2250, 2017
8. Clark RA, Mostoufi-Moab S, Yasui Y, et al: Predicting acute ovarian failure in female survivors of childhood cancer: a cohort study in the Childhood Cancer Survivor Study (CCSS) and the St Jude Lifetime Cohort (SJLIFE). Lancet Oncol 21:436-445, 2020
9. Burns KC, Boudreau C, Panepinto JA: Attitudes regarding fertility preservation in female adolescent cancer patients. J Pediatr Hematol Oncol 28:350-4, 2006
10. Armuand GM, Wettergren L, Rodriguez-Wallberg KA, et al: Desire for children, difficulties achieving a pregnancy, and infertility distress 3 to 7 years after cancer diagnosis. Support Care Cancer 22:2805-12, 2014
11. van Dijk M, van den Berg MH, Overbeek A, et al: Reproductive intentions and use of reproductive health care among female survivors of childhood cancer. Hum Reprod 33:1167-1174, 2018
12. Gorman JR, Drizin JH, Mersereau JE, et al: Applying behavioral theory to understand fertility consultation uptake after cancer. Psychooncology 28:822-829, 2019
13. Zebrack BJ, Casillas J, Nohr L, et al: Fertility issues for young adult survivors of childhood cancer. Psychooncology 13:689-99, 2004
14. Gorman JR, Bailey S, Pierce JP, et al: How do you feel about fertility and parenthood? The voices of young female cancer survivors. J Cancer Surviv 6:200-9, 2012
15. Sandheinrich T, Wondmeneh SB, Mohrmann C, et al: Knowledge and perceptions of infertility in female cancer survivors and their parents. Support Care Cancer 26:2433-2439, 2018
16. Reinmuth S, Liebeskind AK, Wickmann L, et al: Having children after surviving cancer in childhood or adolescence - results of a Berlin survey. Klin Padiatr 220:159-65, 2008

17. Fertility preservation in patients undergoing gonadotoxic therapy or gonadectomy: a committee opinion. Fertil Steril 112:1022-1033, 2019

18. Daniel LC, Sabiston CM, Pitock M, et al: Fertility Preservation in Young Adults: Prevalence, Correlates, and Relationship with Post-Traumatic Growth. J Adolesc Young Adult Oncol, 2020

19. Quinn GP, Block RG, Clayman ML, et al: If you did not document it, it did not happen: rates of documentation of discussion of infertility risk in adolescent and young adult oncology patients' medical records. J Oncol Pract 11:137-44, 2015

20. Flink DM, Sheeder J, Kondapalli LA: Do Patient Characteristics Decide if Young Adult Cancer Patients Undergo Fertility Preservation? J Adolesc Young Adult Oncol 6:223-228, 2017

21. Logan S, Perz J, Ussher JM, et al: Systematic review of fertility-related psychological distress in cancer patients: Informing on an improved model of care. Psychooncology 28:22-30, 2019

22. Gilleland Marchak J, Elchuri SV, Vangile K, et al: Perceptions of Infertility Risks Among Female Pediatric Cancer Survivors Following Gonadotoxic Therapy. Journal of pediatric hematology/oncology 37:368-372, 2015

23. Canada AL, Schover LR: The psychosocial impact of interrupted childbearing in long-term female cancer survivors. Psycho-oncology 21:134-143, 2012

24. Gorman JR, Malcarne VL, Roesch SC, et al: Depressive symptoms among young breast cancer survivors: the importance of reproductive concerns. Breast cancer research and treatment 123:477-485, 2010

25. Carter J, Raviv L, Applegarth L, et al: A cross-sectional study of the psychosexual impact of cancer-related infertility in women: third-party reproductive assistance. Journal of cancer survivorship : research and practice 4:236-246, 2010

26. Lehmann V, Keim MC, Nahata L, et al: Fertility-related knowledge and reproductive goals in childhood cancer survivors: short communication. Hum Reprod 32:2250-2253, 2017

27. Cherven BO, Mertens A, Wasilewski-Masker K, et al: Infertility Education: Experiences and Preferences of Childhood Cancer Survivors. J Pediatr Oncol Nurs 33:257-64, 2016

28. Meacham LR, Williamson-Lewis R, Cherven BO, et al: Educational Intervention to Address Infertility-Related Knowledge Gaps Among Adolescent and Young Adult Survivors of Childhood Cancer. Journal of adolescent and young adult oncology:10.1089/jayao.2019.0156, 2020

29. Lehmann V, Chemaitilly W, Lu L, et al: Gonadal Functioning and Perceptions of Infertility Risk Among Adult Survivors of Childhood Cancer: A Report From the St Jude Lifetime Cohort Study. J Clin Oncol 37:893-902, 2019

30. Lehmann V, Nahata L, Ferrante AC, et al: Fertility-Related Perceptions and Impact on Romantic Relationships Among Adult Survivors of Childhood Cancer. J Adolesc Young Adult Oncol 7:409-414, 2018

31. Ferrante AC, Gerhardt CA, Yeager ND, et al: Interest in Learning About Fertility Status Among Male Adolescent and Young Adult Survivors of Childhood Cancer. J Adolesc Young Adult Oncol 8:61-66, 2019

32. Kim J, Mersereau JE: A pilot study about female adolescent/young childhood cancer survivors' knowledge about reproductive health and their views about consultation with a fertility specialist. Palliative & supportive care 13:1251-1260, 2015
33. Benedict C, Thom B, D NF, et al: Young adult female cancer survivors' unmet information needs and reproductive concerns contribute to decisional conflict regarding posttreatment fertility preservation. Cancer 122:2101-9, 2016
34. Panjwani AA, Marín-Chollom AM, Pervil IZ, et al: Illness Uncertainties Tied to Developmental Tasks Among Young Adult Survivors of Hematologic Cancers. J Adolesc Young Adult Oncol 8:149-156, 2019
35. Newton K, Howard AF, Thorne S, et al: Facing the unknown: uncertain fertility in young adult survivors of childhood cancer. J Cancer Surviv, 2020
36. Hansen KR, Hodnett GM, Knowlton N, et al: Correlation of ovarian reserve tests with histologically determined primordial follicle number. Fertil Steril 95:170-5, 2011
37. Children's Oncology Group: Long-Term Follow-Up Guidelines for Survivors of Childhood, Adolescent, and Young Adult Cancers, Version 5.0. Monrovia, CA, Children's Oncology Group, 2018
38. Nilsson J, Jervaeus A, Lampic C, et al: 'Will I be able to have a baby?' Results from online focus group discussions with childhood cancer survivors in Sweden. Human reproduction (Oxford, England) 29:2704-2711, 2014
39. Benedict C, McLeggon JA, Thom B, et al: "Creating a family after battling cancer is exhausting and maddening": Exploring real-world experiences of young adult cancer survivors seeking financial assistance for family building after treatment. Psychooncology 27:2829-2839, 2018
40. Arnett JJ: Emerging adulthood. A theory of development from the late teens through the twenties. Am Psychol 55:469-80, 2000
41. O'Connor AM, Jacobsen MJ, Stacey D: An evidence-based approach to managing women's decisional conflict. J Obstet Gynecol Neonatal Nurs 31:570-81, 2002
42. O'Connor AM: Ottawa Decision Support Framework to Address Decisional Conflict, 2006
43. Woodard TL, Hoffman AS, Covarrubias LA, et al: The Pathways fertility preservation decision aid website for women with cancer: development and field testing. J Cancer Surviv 12:101-114, 2018
44. Peate M, Meiser B, Cheah BC, et al: Making hard choices easier: a prospective, multicentre study to assess the efficacy of a fertility-related decision aid in young women with early-stage breast cancer. Br J Cancer 106:1053-61, 2012
45. O'Connor AM: Validation of a decisional conflict scale. Med Decis Making 15:25-30, 1995
46. Children's Oncology Group: Long-Term Follow-Up Guidelines for Survivors of Childhood, Adolescent, and Young Adult Cancers Version 5.0; Summary of Cancer Treatment Version, 2018
47. Centers for Disease Control and Prevention, National Center for Health Statistics: National Survey of Family Growth, 2015
48. Arnett JJ: Conceptions of the Transition to Adulthood: Perspectives From Adolescence Through Midlife. Journal of Adult Development 8:133-143, 2001

49. Sharon T: Constructing Adulthood: Markers of Adulthood and Well-Being Among Emerging Adults. Emerging Adulthood 4:161-167, 2015

50. Baggio S, Iglesias K, Studer J, et al: An 8-Item Short Form of the Inventory of Dimensions of Emerging Adulthood (IDEA) Among Young Swiss Men. Eval Health Prof 38:246-54, 2015

51. Faas C, McFall J, Peer J, et al: Emerging adulthood MoA/IDEA-8 scale characteristics from multiple institutions Emerging Adulthood 8:259-269, 2020

52. Balthazar U, Fritz MA, Mersereau JE: Fertility preservation: a pilot study to assess previsit patient knowledge quantitatively. Fertil Steril 95:1913-6, 2011

53. Meneses K, McNees P, Azuero A, et al: Development of the Fertility and Cancer Project: an Internet approach to help young cancer survivors. Oncol Nurs Forum 37:191-7, 2010

54. Meneses K, McNees P, Azuero A, et al: Evaluation of the Fertility and Cancer Project (FCP) among young breast cancer survivors. Psycho-oncology 19:1112-1115, 2010

55. Quinn GP, Knapp C, Murphy D, et al: Congruence of reproductive concerns among adolescents with cancer and parents: pilot testing an adapted instrument. Pediatrics 129:e930-6, 2012

56. Quinn GP, Murphy D, Knapp CA, et al: Coping Styles of Female Adolescent Cancer Patients with Potential Fertility Loss. J Adolesc Young Adult Oncol 2:66-71, 2013

57. Moura-Ramos M, Gameiro S, Canavarro MC, et al: Assessing infertility stress: re-examining the factor structure of the Fertility Problem Inventory. Hum Reprod 27:496-505, 2012

58. Zurlo MC, Cattaneo Della Volta MF, Vallone F: Factor structure and psychometric properties of the Fertility Problem Inventory-Short Form. Health Psychol Open 4:2055102917738657, 2017

59. Gorman JR, Su HI, Pierce JP, et al: A multidimensional scale to measure the reproductive concerns of young adult female cancer survivors. J Cancer Surviv 8:218-28, 2014

60. Gorman JR, Pan-Weisz TM, Drizin JH, et al: Revisiting the Reproductive Concerns After Cancer (RCAC) scale. Psychooncology 28:1544-1550, 2019

61. Centers for Disease Control and Prevention NCfHS: National Health and Nutrition Examination Survey, 2019

62. Sandelowski M: What's in a name? Qualitative description revisited. Res Nurs Health 33:77-84, 2010

63. Neergaard MA, Olesen F, Andersen RS, et al: Qualitative description - the poor cousin of health research? BMC Med Res Methodol 9:52, 2009

64. Sandelowski M: Whatever happened to qualitative description? Res Nurs Health 23:334-40, 2000

65. Williamson R, Meacham L, Cherven B, et al: Predictors of successful use of a web-based healthcare document storage and sharing system for pediatric cancer survivors: Cancer SurvivorLink. J Cancer Surviv 8:355-63, 2014

66. Kim J, Mersereau JE, Su HI, et al: Young female cancer survivors' use of fertility care after completing cancer treatment. Supportive care in cancer : official journal of the Multinational Association of Supportive Care in Cancer 24:3191-3199, 2016

67. Patton M: Qualitative Research & Evaluation Methods. (ed 4th). Thousand Oaks, CA, Sage Publications, 2015

68. Miles M, Huberman, A., & Saldana, J.,: Qualitative Data Analysis: A Methods Sourcebook (ed 3rd). Thousand Oaks, CA, Sage Publications 2014

69. Yin R: Case Study Research: Design and Methods (ed 5th). Thousand Oaks, CA, Sage Publications, 2014
